# Apricot Kernel: Bioactivity, Characterization, Applications, and Health Attributes

**DOI:** 10.3390/foods11152184

**Published:** 2022-07-22

**Authors:** Mansoor Ali Akhone, Aarti Bains, Mansuri M. Tosif, Prince Chawla, Melinda Fogarasi, Szabolcs Fogarasi

**Affiliations:** 1 Department of Food Technology and Nutrition, Lovely Professional University, Phagwara 144411, Punjab, India; mansoorali8494@gmail.com (M.A.A.); tosifmansuri444@gmail.com (M.M.T.); 2 Department of Microbiology, Lovely Professional University, Phagwara 144411, Punjab, India; aarti05888@gmail.com; 3 Department of Food Engineering, University of Agricultural Sciences and Veterinary Medicine of Cluj-Napoca, Calea Mănăstur 3–5, 400372 Cluj-Napoca, Romania; 4 Faculty of Chemistry and Chemical Engineering, Department of Chemical Engineering, Babeş-Bolyai University, 11 Arany Janos Street, 400028 Cluj-Napoca, Romania; szabolcs.fogarasi@ubbcluj.ro; 5 Interdisciplinary Research Institute on Bio-Nano-Sciences, Babeş-Bolyai University, 42 Treboniu Laurian Street, 400271 Cluj-Napoca, Romania

**Keywords:** apricot, apricot kernel, food application, pharmaceutical applications

## Abstract

Apricot kernel, a by-product of apricot fruit, is a rich source of proteins, vitamins, and carbohydrates. Moreover, it can be used for medicinal purposes and the formation of food ingredients. Several techniques have been adopted for the extraction of bioactive compounds from the apricot kernel such as solvent extraction, ultra-sonication, enzyme-assisted, microwave-assisted, and aqueous extraction. Apricot kernels may help to fight against various diseases such as cancer and cancer immunotherapy, as well as reduce blood pressure. Additionally, the kernel is famous due to its diverse industrial applications in various industries and fields of research such as thermal energy storage, the cosmetic industry, the pharmaceutical industry, and the food industry. Especially in the food industry, the apricot kernel can be used in the preparation of low-fat biscuits, cookies, cakes, and the fabrication of antimicrobial films. Therefore, in this review article, the bioactivity of the apricot kernel is discussed along with its chemical or nutritional composition, characterizations, and applications.

## 1. Introduction

Over the past years, the Rosaceae family has attained high demand in several industries due to its potential therapeutic properties and industrial applications [1]. The apricot tree has a height of around 8–12 m and bears yellowish small-sized fruit. The apricot tree contains five petals of white flowers with leaves of oval shape. The kernel is an organic product that positively impacts human health and is often considered an unwanted part of the fruit [2]. Apricots possess sweet edible kernels. However, a few varieties are bitter and “inedible”. According to the reports on the protection of plant varieties and farmer’s welfare, the Government of India, there are different varieties of apricots with sweet edible kernels such as Hangole, Sirkhant, Gylchima, Chharb, Ston chulli, Pangpong, Zanchuli, Razacho, Mumuri, Yakchey Karpo, Channya Narmo, Khabulo, Marpo chulli, and Stingsos.Some bitter apricot kernel varieties are also found, i.e., Khantey chuli, Khantey Halman, Nimra, Scho, Stingchos, Khantey Phatting, Khusta, Khanchuli, Thopchi, and Stangyachuli. Apricot (*Prunus armeniaca* L.) originated in China, and later it was introduced to various parts of Central Asia [3]. Globally, annual fresh apricot production was 4,260,466 tonnes, whereas the production of dried apricot was 162,635 tonnes in 2018 and 2019 as per the reports of FAO 2019. Turkey is the world’s leading producer of apricots (127,328 tons) and produces approximately 22.9% of the world’s fresh apricots and nearly 68% of the world’s dried apricots [4]. According to available literature, it has been concluded that apricot kernels can also be utilized for thermal energy storage [5], traditional medicine [6], production or maintaining the quality of oils [7], cosmetic products [8,9], food products [10], and fabrication of antimicrobial film [11]. Especially in the food industry, apricot kernels along with skins are used in the formulation of bakery and confectionery products [12]. Depending upon the different varieties, apricot seeds contain cyanogenic glycoside amygdalin. It is commonly found in the bitter apricot kernel, cherry, apple seed, and almond. Several reports revealed that apricot kernel has been used for treating hypertension, chronic inflammation, cancer, and other reaction diseases and for the treatment of migraine. In addition, amygdalin also improves cerebral function [13]. Amygdalin (present in the skin of the kernel) plays an imperative role against cancer [14]. According to a systematic review, the amygdalin compound presented pharmacological activities of anti-tumor, anti-fibrotic, anti-inflammatory, analgesic, immunomodulatory, and anti-atherosclerosis, ameliorating the digestive system and reproductive system, improving neurodegeneration and myocardial hypertrophy, as well as reducing blood glucose. In addition, studies revealed that amygdalin’s toxicity was caused by its poisonous decomposite product of benzaldehyde and hydrogen cyanide after oral ingestion; the toxicity of the intravenous administration route was far less than the oral route, and it can be avoidable with an oral dose ranging from 0.6 to 1 g per day [15]. Moreover, Alajil et al. [16] reported that the maximum tolerance of amygdalin by human intravenous injection is approximately 0.07 g/kg, and interestingly, numerous studies revealed that bitterness is one of the major causes that restrict the industrial application and nutritional value of wild apricot kernels [5]. Despite the several benefits of the fruit, the apricot kernel comprises potential bioactive components and nutrients including carotenoids, carbohydrates, vitamins, phenols, terpenoids, esters, and volatile compounds [7]. The apricot kernel is also considered a promising ingredient in the health sector as it has anti-cancer, antioxidant, antimicrobial, antiasthma, inflammatory, atherosclerotic, anti-analgesic, and anti-hyperlipidemia properties [7]. They hold enormous properties in several industries inducing cosmetic [17], pharmaceutical [7], and food industries [18] due to their low cost and eco-friendly nature. However, several techniques have been adopted for the extraction of bioactive compounds from the apricot kernel such as solvent extraction, ultrasonication, enzyme-assisted, microwave-assisted, and aqueous extraction [19]. Furthermore, the waste valorization of kernels from apricot has achieved greater economic benefits and reduced waste disposal problems as it can be converted into value-added products [20]. The composition of apricot oil and nutrients are highly dependent upon the fruit variety, origin, maturity stage, and climatic conditions [21]. The extraction of bioactive compounds from the wild apricot kernel is a major challenge for research [22]. Therefore, this review article emphasizes the bioactivity, characterization, and various applications of the apricot kernel. Additionally, the therapeutical properties of the apricot kernel have been discussed with schematic diagrams and mechanisms.

## 2. Nutritional and Chemical Composition of Apricot Kernel

**Figure 1:**
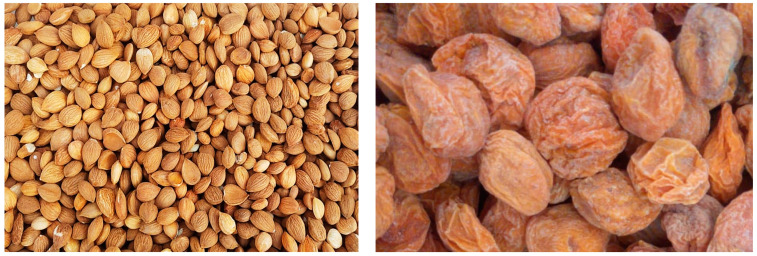
Apricot kernel seed and fruit identification.

Apricot is considered a delicious fruit and can be grown in various parts of the world; it is also famous due to its potential nutritional and chemical composition. Identification features of apricot seed and fruit are represented in Figure 1. The kernel contains the toxic phenylalanine-derived cyanogenic glycoside amygdalin, accompanied by minor amounts of prunasin, which is a precursor of the diglucoside amygdalin, a β-D-monoglucoside of R-mandelonitrile. Upon tissue disruption, amygdalin and its precursor prunasin are degraded by specific β-glucosidases, resulting in the release of toxic hydrogen cyanide, which serves as a defense mechanism against generalist herbivores [5]. According to the literature, the apricot kernel comprises various polyphenolic compounds such as anthocyanin, phenolics, flavonoids, carotenoids [20,21,22,23,24,25], Vitamin E [26], and quality proteins (Table 1) [23].

Protein extract of apricot kernels can be used for the preparation of transglutaminase-induced gel, which can also be used for the delivery of sensitive compounds into functional foods, dietary supplements, and pharmaceutical products [43,44]. It may help to cure several diseases by reducing blood pressure along with treating cancer, and cancer immunotherapy [45]. In this regard, Chaouali et al. [45] described the hydrocyanide (HCN) content in the apricot kernel as ranging from 9.18–12.53 mg. Anthocyanin from different varieties of apricot seed kernel was extracted by Al Juhaimi et al. [20]. They revealed that anthocyanin content was significantly increased (0.38–0.84 mg) with increasing microwave power (720 W). Flavonoids are widely used as anti-cancer, neuroprotective, anti-tumor, anti-proliferative agents, and anti-angiogenic antimalarial agents. They can be classified based on their oxidation of carbon rings, degree of unsaturation, and chemical structure, whereas flavonoids are divided into different subgroups including flavanones, flavans, anthocyanin, and isoflavonoids. It helps in preventing cardio-metabolic disorders and many other diseases such as cognitive performance of aging, colorectal cancer, promoting cardiac wellness, supporting weight loss, and preventing diabetes. Phenol compounds were extracted by roasting the apricot kernels in the microwave. The average total phenol content in the apricot kernel was ranging from 36–72 mg [39]. Furthermore, the antioxidant activity in the apricot kernel after roasting at 360 and 540 W increased rapidly according to the studies performed by Al Juhaimi et al. [20]. It has been believed that polyphenols provide various health benefits with the help of different mechanisms such as the elimination of free radicals, the protection and regeneration of other dietary antioxidants and vitamins, and the chelation of pro-oxidant metals [46]. Apricot kernels are a nutritionally remarkable source of proteins, which are the second richest component of their weight. Protein content in the apricot kernel range from 14.6 to 27.1%. [42].

**Figure 2:**
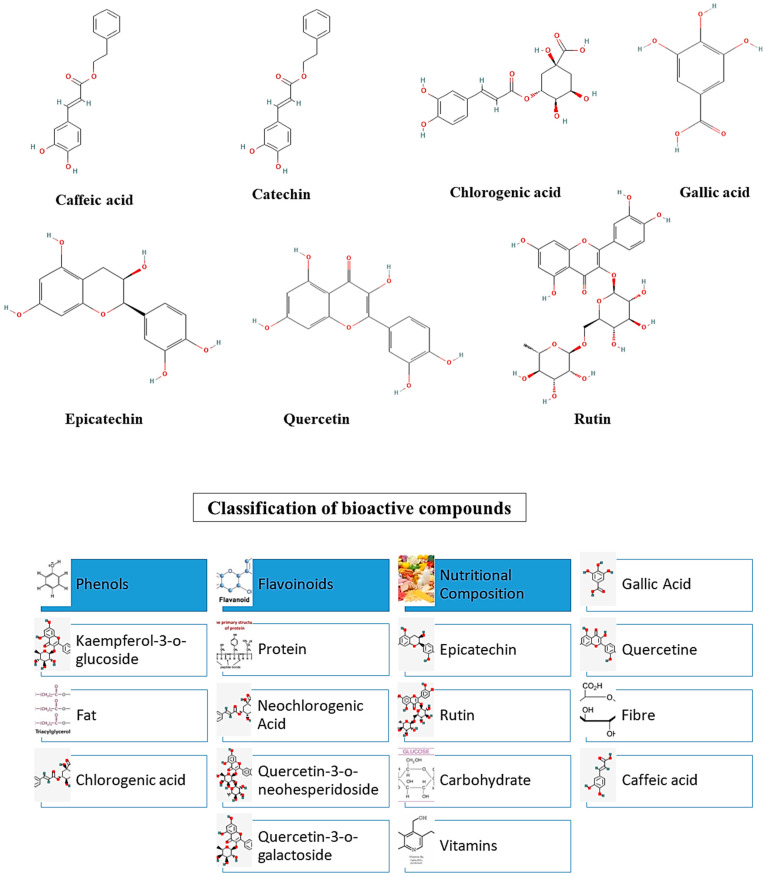
Chemical structures of different bioactive compounds present in the apricot kernel and their classification.

Several studies have been conducted to determine the chemical and nutritional properties of the apricot kernel and its health attributes. It has been also stated that the kernel is a promising ingredient for the formulation of nutraceutical, pharmaceutical, and food products due to its distinctive properties [36]. For example, the antioxidant potential of the apricot kernel can help against various chronic diseases such as cancer stroke, diabetes, etc. Apart from this, it can act as a dietary fiber and has a high number of polyphenolic extracts. The high amount of dietary fiber has shown to have a great impact on human health as it can be beneficial for gut health and cholesterol absorption and has anti-obesity and anti-diabetic properties. In this context, few studies have revealed that phenolic compounds such as caffeic acid, gallic acid, epicatechin, rutin, and chlorogenic acid exist in the apricot kernel [39]. The presence of flavonoid compounds such as quercetin, catechins, and chlorogenic acid exists in the apricot seed, which results in lower oxidative stress. It also consists of anthocyanins, which protect the skin by lowering the risk of sunburn and wrinkles. Furthermore, the protein substance present in the apricot kernel increases the antioxidant activity, which is required in food. Several studies have revealed that the apricot kernel consists of various types of antioxidants such as lutein, beta carotene, and zeaxanthin, which also help in fighting against free radicals [21]. The chemical structures of various polyphenolic compounds and their classifications are shown in Figure 2. 

## 3. Extraction of Bioactive Compounds from Apricot Kernel

Apricot kernel has been known to possess a wide variety of biologically active components. Generally, microwave extraction, supercritical pressurized liquid extraction, ultrasound-assisted extraction, and microwave solvent extraction methods have been employed for the extraction of the different classes of bioactive compounds [47,48,49,50,51,52,53,54,55]. Different techniques adapted to extract the bioactive compounds from the apricot kernel are represented in Table 2.

### 3.1. Two-Phase Extraction Method

**Figure 3:**
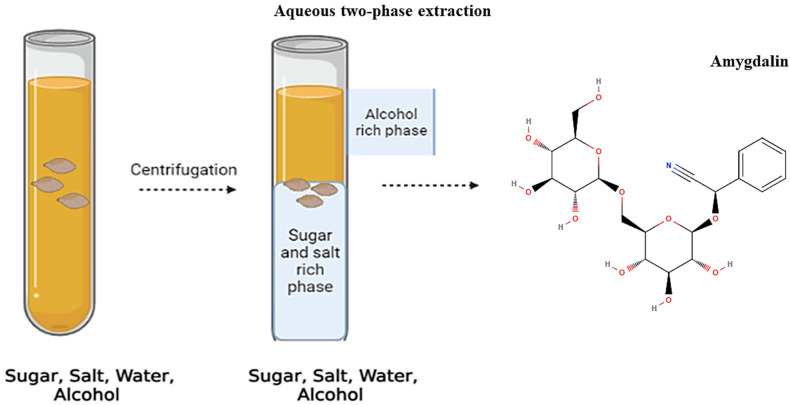
A two-phase extraction method for extraction of amygdalin.

The two-phase extraction system is collected form of two different immiscible substances such as polymer or mixing polymers with salt; they are water-soluble in a certain concentration. It has been well known as a useful technique for the separation and purification of biomolecules, such as proteins and antibodies [49]. A selective exchange taking place between the definite ligands in one of the polymeric phases of the system and the barrier of the desired protein, such as a biomolecule or cellular fragment, towards the modified polymer-rich phase can be achieved [50]. The system is a liquid-liquid fractionation technique that has gained interest because of its great potential for the separation, extraction, and enrichment of proteins, purification membranes, enzymes, nucleic acids, viruses, and other biomolecules and industrial applications [51]. In this context, Zhang et al. [14] used a two-phase method for the extraction process in which the bioactive compound was amygdalin. The main purpose of this method was to recycle the amygdalin (90.37%) as shown in Figure 3. This method is considered a better extraction technique than the conventional extraction technique because it is low-cost, environment-friendly, and capable of continuous operation for various kinds of experiments such as the concentration and purification of biomolecules [51].

### 3.2. Solvent Extraction Method

It is one of the most used methods, also known as the liquid–liquid extraction method used to separate the compounds based on their relative solubilities. According to the literature, apricot kernel oil can be extracted by different extraction techniques such as supercritical fluid extraction, cold pressing, enzyme-assisted extraction, solvent (n-hexane) extraction, or a combination of these methods. On a commercial scale, vegetable oils are usually produced by organic solvent extraction as this process recovers high yields [52]. Consequently, extraction of bioactive compounds by freeze-drying techniques has attained high demand in industries due to its low cost of maintenance. A bioactive compound (phenolic compound) was extracted by freeze-drying and a 6–9% yield was obtained by Gaya et al. [52]. In this study, initially, acetone, acetyl chloroform, and ethanol solvents were used to extract the phenolic and flavonoid compounds. After, the extraction sample was freeze-dried for further applications. Solvent extraction is considered an injurious method due to the utilization of chemicals; due to this reason, solvent extraction is high.

### 3.3. Cold Press Extraction Method

Cold pressing is a mechanical extraction technique also known as mechanical separation used to extract bioactive compounds. Cold pressing is now becoming a more in-demand method as this process does not need any organic solvents or heat and it enables the retention of appreciable contents of minor bioactive components such as phytosterols, and free radical scavengers, phenols, and in the recovered oils [53]. On other hand, this technique has some limitations due to its high cost and maintenance. Bioactive compounds (Amygdalin and Tocopherols) were extracted by cold press extraction technique, which gave a yield of (5.84–62.73%) [54,55]. Apricot kernel constitutes some effective polyphenols such as 3- and 5-feruloyl quinic acid, 3 caffeoylquinic, quercetin-3-xyloside, quercetin-3-rhamnoside, and catechin as confirmed by high-performance liquid chromatography (HPLC) [53]. For instance, the oil of apricot kernels was extracted by the ultrasound extraction method. As compared to the other conventional extraction techniques such as cold press, freeze-drying, two-phase, and steam distillation, this process can complete the extraction process within a few minutes with reduced consumption of solvents and water, high reproducibility, greater purity of the end products, and consuming only a portion of the energy, which is necessary for the process [54]. This method has the advantage that it is not restricted by the moisture content of the products, or by the type of solvent used. It is well known that mass transfer methods are enhanced by exposure to ultrasound power [56]. The apricot kernels were ground into powder, and extraction was carried out in an ultrasonic bath with a frequency of 40 kHz. Gas chromatography-mass spectrometry (GCMS) is an advanced analytical technique effectively used for the characterization of extracted bioactive compounds or oils from various food products. For example, the composition of fatty acid in the apricot kernel was estimated using the GCMS, and the gastroprotective properties were also studied [12]. Herein, results of GCMS showed that apricot kernel oil is rich in several fatty acids including palmitic acids (3.11%), stearic acid (8.38%), eicosadienoic acid (15.45%), linoleic acid (16.58%), and oleic acid (56.48%) [57]. 

## 4. Measured Characteristics of Apricot Kernel Using Different Techniques

### 4.1. Fourier Transform Infrared Spectroscopy

Fourier-transform infrared spectroscopy (FT-IR) is an analytical technique used to assess the chemical bonds or functional groups present in an apricot kernel or any other component by generating an infrared absorption spectrum [58]. FT-IR spectroscopy has been utilized to test the structural dynamics, structural composition, conformational changes (effect of temperature, binding, and pH), structural stability, and aggregation of proteins. Similarly, Alatabe et al. [59] utilized this method for testing the structural composition, structural stability, structural dynamics, conformational changes, and aggregation of proteins. Different studies revealed that the apricot kernel is constituted by C=O, CH3, OH, C-O, CH, and C-O-H groups. Likewise, OH- stretching (water), Aliphatic isonitrile –N=C stretching, C= C stretch or C=O stretch, Combination N-H stretching, Combination O-H stretching, Methylene scissor, Secondary amide N-H bonding, P-Cl stretching, C-C stretch of starch, and P-S stretching were attributed at 3751.67, 2156.49, 1651.12, 2011.82, 2011.82, 1456.30, 1541.18, 522.73, 1047.38, and 441.71 cm^−1^, respectively [60]. Janković et al. [24] characterized apricot kernel shells by FTIR. In their study, they stated that a wavelength range between 1800–800 cm^−1^ is considered a fingerprinting area, whereas -CH and -OH stretching vibrations were confirmed in the first region (4000–2300 cm^−1^). Wavelengths 2925, 2950, and 3010 were assigned to –CH3, CH2, –CH, and –OH stretching vibration, respectively. In another study, a wavelength of 1743.71 cm^−1^ was attributed to aldehyde and ketone and C=O stretching, followed by O-H stretching confirmed at high intense peaks of 2922.25 cm^−1^ and 2852.81 cm^−1^, and C-H stretching vibrations were observed due to (RCOOH) group [61]. Furthermore, Fungjay et al. [60] used FTIR to study the functional group of the apricot green shell where the sample was treated with 15% H_3_PO_4_, and 15% H_3_PO_4_ showed a narrow peak at 1037 cm^−1^, which represented C-OH vibration. In addition to this, OH and C-H bonds were observed at a peak of 2897 and 3321 cm^−1^. The peaks between 1300–2000 cm^−1^ were lost after the treatment with H_3_PO_4._

### 4.2. Scanning Electron Microscopy (SEM)

A Scanning Electron Microscope (SEM) is a type of electron microscope that scans surfaces of microorganisms using a beam of electrons moving at low energy to focus and scan specimens [61]. The development of electron microscopes was due to the inefficiency of the wavelength of light microscopes. Electron microscopes have very short wavelengths in comparison to the light microscope, which enables better resolution power [11]. It uses a focused beam of high-energy electrons to generate a change of signals at the surface of solid specimens and is considered one of the most versatile methods used to study surface morphology, microstructure, and physical (size and shape) properties of components [61]. Apart from this, the presence of protein is shown by the formation of small cracks on the surface, whereas the irregular and polygonal shows the presence of starch molecules in the sample and also demonstrates the planning of starch granules and protein networks in the matrix. SEM was conducted at a magnification of 1000x and 1200x [60]. After using a scanning electron microscope, the apricot pomace cell was dislocated with a small presence of the intact cell. From this perspective, Ali et al. [62] studied the characterization of starch-based composite films of the apricot kernel where the results showed confirmation of the homogeneous dispersion of the shell particle can be found in both normal and polarized images and the smooth surface of the starch-based film while the shell particles can be spotted in both normal and polarized images. 

### 4.3. High-Performance Liquid Chromatography (HPLC)

The constituent of polyphenol in apricot pomace was extracted by using different methods. Rutin, catechin, and epicatechin are considered major extracts that exist in apricot pomace [61]. However, epicatechin was only detected in infrared extract (4 µg/g DM) whereas rutin was present in all the experimented extracts, and catechin was noticed during the extraction process using microwave-assisted (2.1 µg/g DM), Ultrasonication (1.5 µg/g DM), and Infra-radiation (3.1 µg/g DM) [63]. Similarly, catechin and epicatechin gave higher yield infrared techniques as compared to ultrasound and microwave techniques. Rutin, Catechin, and Epicatechin were proven to be strong antioxidant molecules and have a synergistic effect, which contributes to high biological activities [41]. Based on this technique, the amygdalin in apricot kernel content was measured, which was 129.34 ± 0.99 mg/g [63]. Moreover, the HPLC method was also used to analyze the amygdalin content in debittered water when the apricot kernels were taken out from the water for tasting and one typical chromatogram. The results showed that the chromatogram has an excellent resolution of amygdalin in the debittered water; the result of amygdalin content was calculated according to the curve standard. Amygdalin content in debittered water increased with the addition of debittering water at a temperature of 50 to 70 °C at a given temperature. On the other hand, it decreased with the increase when the time and temperature of debittering water were fixed [64]. Therefore, the time to obtain the apricot kernels without bitterness was 8, 5, and 6 h. for the temperature of 50, 60, and 70 °C, respectively. It also shows that time and temperature have an important role to play in debittering the apricot kernel [61]. Results revealed that the content of the most predominant compound (protocatechuic aldehyde) is 1.24 mg/g (wet base) and 2.69 mg/g (dry base), respectively [65]. 

### 4.4. X-ray Diffraction (XRD)

X-ray diffraction (XRD) is an influential nondestructive technique for characterizing crystalline materials. This method provides information on phases, preferred crystal orientations (texture), structures, and other structural parameters, such as size, strain, average grain, crystal defects, and crystallinity. X-ray diffraction peaks are formed by constructive interference of a monochromatic beam of X-rays scattered at exact angles from each set of lattice planes in a sample. The peak strengths are determined by the supply of atoms within the lattice [66]. XRD was used to analyze the apricot kernel shell. According to various literature, the peak was observed around 20 to 22 °C, the main evidence of which was the cellulose as a component of the apricot kernel shell [67,68,69]. Moreover, Ali et al. [62] observed the crystallization behavior of the apricot kernel and nut where the normal peaks were observed at 2θ = 14.76 and 24.35 °C, whereas the diffraction peaks were identified at 2θ = 17.65 and 20.40°. With the incorporation of both the shell and kernel, the intensity and characteristic diffraction peak of starch increased slightly to 2θ = 17.65 °C, which also specifies that the crystalline structure of starch is not only conserved but also showed the interaction of filler with the matrix. Furthermore, the optical microscopic images also verify the crystalline structure and great depression of shell particles in the starch matrix. 

## 5. Application of Apricot Kernel

### 5.1. Application of Apricot Kernel Flour in the Food Industry

Apricot kernel flour has been reported to be a good source of minerals, protein, bioactive compounds, and fiber, and it has been mainly used in the bakery industry for the protection of products. On the other hand, there is an increasing demand of consumers for foods that do not fulfill the basic need of nutrition but helps in preventing various disease and other curing roles [43]. Apricot kernels are mainly used in the production of oils and benzaldehyde; the kernels are also added to bakery products either whole or grounded and also consumed as an appetizer [32]. According to, Kopčeková et al. [67] apricot kernel flour can be used in the preparation of biscuits, cookies, cakes, etc. Kernels showed a positive impact on yogurt to improve sensory properties [44]. The apricot kernel alleviates the risk of free radicals, which cause oxidative damage to the living cells and result in common degenerative disorders such as cardiovascular diseases and cancer [7]. The kernel powder was defatted and used as a source of protein in yogurt and ice cream in the ratio of 10–40% and 10–50%, respectively [63]. Moreover, the stability of protein was higher in apricot kernel products. From this result, they came to know that apricot kernel powders up to 30% can be substituted with ice cream [40]. Similarly, in the case of yogurt, the addition of apricot kernel powder led to a decrease in the lactic acid bacteria count, pH, and acetaldehyde value [18]. It was concluded that skim milk could be substituted up to a level of 20% in the yogurt [70]. In a study, the quality of stirred yogurt was improved by adding apricot kernel powder. Cow milk was substituted with 1% of apricot kernel powder. It was noticed that with the addition of apricot kernel powder the titratable acidity, ash content, and total solids and protein content increased [44]. However apricot kernel cake has been utilized as a good source of protein in animal fodder [5].

### 5.2. Application of Apricot Kernel in Pharmaceuticals:

Apricot kernels are being used as a medicine in pharmaceutical industries as they consist of Amygdalin, which has been used to prevent diseases such as migraine, constipation, asthma, and hypertension. They have also been used to treat coughs and improve cerebral functions [69]. A disease-preventing black plum apricot kernel is used for suppressing asthma, cough, thrombosis, and relaxing cough. The kernel seed is also used for the preparation of Chinese Guangmo moon cakes, which are used for the treatment of diseases such as chronic bronchitis, pulmonary tuberculosis, etc. [69]. Over the past few years, the market value of apricot kernel in pharmaceutical industries has increased rapidly in various developed and developing countries as the kernel is used in the preparation of various medicines, which are used for the treatment of cardiovascular diseases, antimutagenic, antitussive, anti-inflammatory, antimicrobial, cancer, etc. [71]. Extract of the apricot kernel can be effectively used as an antifungal ingredient for the prevention of skin and health issues. Apricot oil can promote blood circulation and release pain and inflammation. Additionally, several commercial products are already available in the market [72]. Moreover, apricot kernel flour has been used for the preparation of herbal tea for patients suffering from diseases such as dry mouth, disturbed sleep, anorexia, etc. [73]. Apart from this, the apricot kernel has also been used for the treatment of skin diseases such as acne vulgaris, dandruff, and furuncle due to its antioxidant, antimicrobial, antimicrobial anti-inflammatory, and wound-healing properties. It has also been used to fight free radicals and promotes skin barrier homeostasis [73]. 

## 6. Therapeutical Properties of Apricot Kernel

There is great demand for food that meets the basic nutritional needs of the consumers and has a preventive, therapeutic, and gastroprotective role in disease curing [74]. According to Karatas et al. [74], apricot mostly contains phytochemicals, which play an important role in the human body. Similarly, these compounds reduce the risk of free radicals that cause oxidative damage in living cells and common degenerative disorders such as cancer and cardiovascular diseases [75]. Apricot kernel is an excellent vehicle in the field of medicine as it has broad-spectrum applications (anti-cancer, skin diseases, cardiovascular diseases, hemostasis, the release of pain and inflammation, etc.), and the major anti-inflammatory compounds are Acetylcholinesterase, Human 15 Lipoxygenase, Cycloxygenase, Interleukin 6, Prostaglandin, Toll-like receptors, and Tumor necrosis factor alpha [76]. 

### 6.1. Antioxidant Capacity of the Apricot Kernel

The antioxidant potential of apricot has been repeatedly investigated through different in vitro systems by measuring its ability to reduce free radicals and comparing it with standard reference compounds [77]. Apricot kernel contains caffeic acid (2.5 μg/g) and gallic acid (4.1 μg/g), which are used in the preparation of various medicines and treatment of various diseases such as cardiovascular disease, breast cancer, anti-asthmatic, antiseptic, sedative, emetic, laxative, etc. [78]. According to Zhang et al. [65], there are different maturity stages and genotypes; the geographic region is presently based on the variety of apricots. Apricot kernel has been used for centuries as a popular home remedy in China and among the mountain dwellers of the Himalayas. The major antioxidant components present in the bitter apricot kernel are 2,2-azino-bis (3-ethlybenzothaizoline-6-sulfonic acid, 2,2-diphenyl-1-picrylhydrazyl, ferric reducing antioxidant power assay, oxygen radical absorbance capacity assay, and Trolox equivalent. The rich nutritional composition of apricot and apricot kernel, which contain phytonutrients, saccharides, organic acids, minerals, and vitamins, is the main fact for using this fruit in folk medicine [79].

### 6.2. Anti-Cancer

Cancer is one of the most common degenerative diseases in day-to-day life and it is the second cause of death after cardiovascular diseases [80]. In the human body, the immune system plays a significant role in cancer incidence and inflammation [81]. In the present lifestyle, humans need to take high content of fruits and vegetables in their diet, which has been consistently associated with reducing the risk of several types of cancers up to 30 to 40%, which include lung, breast, prostate, and colon cancers [14]. Apricot contains anti-carcinogenic potential compounds, which helps in curing numerous diseases, and intake of apricots and their kernel during the day can reduce gastric mucosal inflammation and helicobacter pylori infection [7].

### 6.3. Cardiovascular Diseases

Apricot provides a significant amount of soluble fiber and insoluble fiber. These dietary fibers are effective in lowering LDL cholesterol [82]. Similarly, in vivo studies in animals showed a major effect of apricot feeding found to be reduced by up to 10–20% disease compared to the control group [79]. Furthermore, after supplementation of apricot to rats, the level of antioxidant capacities such as iron reducing power as total phenolic content, DPPH radical scavenging capacities have increased. Consumption of apricot kernel also improves the proper functioning of platelets in the human body [83].

### 6.4. Hemostasis

Several epidemiological studies have found a negative relationship between dietary flavonoids and flavones and the risk of cardiovascular disease [84]. According to various studies, dietary intake of flavonoids and flavones, which are present in the apricot kernel, reduces the risk of cardiovascular disease [85]. This is due to the effect of these compounds on hemostasis, as flavonoids have been reported to inhibit platelet aggregation in vitro. Similarly raw apricot contains 4.79 mg/100 g flavon-3-ol (+), epicatechin 2.08 mg/100 g, and catechin 5.47 mg/100 g flavon-3- ol (-) epicatechin 2.08 mg/100 g edible flavonol fraction (USDA, 2007), and studies revealed that 2500 µM/L flavonol quercetin and the flavone apigenin significantly inhibited collagen-induced and ADP-induced aggregation in platelet-rich plasma and inhibited platelets by almost 80–97% [86]. On the other hand, flavonoids help red blood cells fight stress caused by oxidation and help protect red blood cells against reactive oxygen species [87].

### 6.5. Contraindication

Intake of apricot kernels in high amounts is not recommended for pregnant or breastfeeding women due to the potential risk of birth effects [68]. The consumption of apricot kernels by pregnant women leads to thyroid disease during the carrying of babies, and they are exposed to cyanide and thiocyanate during pregnancy. Furthermore, the apricot kernel also helps in lowering blood pressure and making supplements [7]. 

### 6.6. Hepatic Steatosis and Kernel as Folk Medicines

Hepatic steatosis mainly leads to the formation of intra-cytoplasmic accumulation of neutral fats in liver tissues and is called fatty liver disease (FLD). Apricot has been effective in curing hepatic steatosis in animal models. The occurrence of this disease in the general population may further lead to steatohepatitis, advanced fibrosis, and cirrhosis [43]. Among them, phenolic compounds show antioxidant properties such as anticarcinogenic, antiplatelet, anti-microbial, anti-ischemic, anti-tumor, anti-allergic, anti-mutagenic, and anti-inflammatory, as well as being effective in alleviating cardiovascular disease [7]. The Apricot kernel is folk medicine that is mainly used by the Chinese in regenerating body fluid, quenching thirst, and detoxifying, whereas the apricot kernel is used for making syrups which help in alleviating cough and other respiratory problems [82,85]. The folk relates apricot as an antipyretic, antiseptic, antispasmodic, antiasthmatic, analgesic, laxative, demulcent, emetic, expectorant, ophthalmic, sedative, vulnerary, tonic, etc. [86].

## 7. Market Value of Apricot Kernel

The apricot kernel is the internal part of the apricot fruit. The color of the kernel is white when it is fresh but it turns into color once the kernel is dried properly [75]. It constitutes a food amount of protein, vitamins, essential minerals, fiber, antioxidants, protein, etc. [65]. Due to increasing awareness of people toward natural ingredients, apricots and their by-products are attractive to various industries and fields of research. As a result, the apricot kernel helps in boosting skin health, improving metabolism, enhancing vision, reducing the risk of diabetes, and maintaining digestive functioning [88]. Therefore, it is well known across the globe to produce various cosmetics (face masks, scrubs, creams, lotions) and food (snacks, oatmeal, muffins, scones, salads, vegan, and animal cruelty-free personal grooming items). Apart from this the utilization of apricot kernels in the health sector, they are also in high demand for Gastroesophageal Reflux Disease, diabetes, obesity, etc. [85]. 

## 8. Future Research Prospectives and Conclusions

The apricot kernel is the seed found inside the apricot pits. Amygdalin present in apricot is a chemical component that helps in fighting against diseases such as cancer and cardiovascular disease. Since amygdalin is reported to be available in rear plant and animal products, it can be the cheapest source of this important component in food industries. The applications of the apricot kernel in various industries such as food, cosmetics, pharmaceuticals, etc. are immense as it is used in making skin and hair products in cosmetic industries. In the food industry, it is used for making cookies, biscuits, and many other products, whereas in the pharmaceutical industry it is used for making medicines. However, sweet apricots are having greater demand than bitter ones because the compound (Amygladin) present in bitter apricot restricts their applications, especially in the food industry. However, different extraction methods have shown excellent efficiency to remove or reduce the bitterness of apricot kernel. Therefore, still, apricot kernel is a great source of research as it has excellent techno-functional and therapeutical properties, especially in the food and pharma industries. The apricot kernel can be considered a potential ingredient in industries due to its cost-effective and eco-friendly nature. 

## Figures and Tables

**Table 1:** Different chemical and nutritional components present in the apricot kernel.

Composition	Amount/100 g	Reference
Protein content	14.6–27.1	[23,24]
Carbohydrate	17.5–35.6	[18,25]
Vitamin E	0.003–0.040	[26]
Vitamin B17	0.003–0.0058	[27]
Mineral (Ca, Fe, P, Na, Mg, Cu, & Mn)	0.0076, 0.0042, 0.0028, 0.0011, 0.003, 0.007, and 0.001	[7,28]
Crude fiber	11.85–13.6	[24,29]
Crude fat Oleic acid	2.1–3 54.1–61.91	[20,30,31] [16]
Linolieic acid Palmatic acid Ash content	25.13–35.81 1.58–2.27 1.3–2.23	[16] [16] [30,32,33]
Moisture content	27.4–38.8	[34,35]
Hydro cyanide	0.009–0.012	[36,37]
Anthocyanin	0.005–0.002	[20,38]
Total phenol content Gallic acid	0.036–0.072 2.1–4.1	[39,40] [41]
Flavonoid content	0.012–0.034	[40,42]
Carotenoid content	0.005–0.012	[40]
Ascorbic acid Caffiec acid	0.010–0.022 1.01–2.5	[39] [41]

**Table 2:** Extraction & Application of Bioactive Compound of Apricot Kernel.

Extraction Method	Bioactive Compound	Yield g/100 g	Applications	References
Two-phase Cold pressing (at 40–120 °C)	Amygdalin	90.37	Application on recycling of amygdalin	[55]
Hot water treatment (at 160 °C)	Amygdalin	0.129	Application in Prevention of keratoconjunctivitis sicca diseases	[51]
Solvent extraction Acetone, acetyl chloroform, and ethanol.	Tocopherols	73.4–94.4	-	[52]
Cold pressing and SC-CO_2_	Amygdalin and Tocopherols	5.84–62.73	Production of oils	[55]
Ultrasonic extraction	Phenolic compounds	40.86–46.01	Helps in enhancing the yield and maintaining the quality of the oils.	[52]
Freeze drying	TPC and TFC	6–9	-	[41]

## Data Availability

Data sharing is not applicable to this article.
